# Twelve Threats of Precision Livestock Farming (PLF) for Animal Welfare

**DOI:** 10.3389/fvets.2022.889623

**Published:** 2022-05-27

**Authors:** Frank A. M. Tuyttens, Carla F. M. Molento, Said Benaissa

**Affiliations:** ^1^Flanders Research Institute for Agriculture, Fisheries and Food (ILVO), Merelbeke, Belgium; ^2^Department of Veterinary and Biosciences, Faculty of Veterinary Medicine, Ghent University, Merelbeke, Belgium; ^3^Animal Welfare Laboratory, Federal University of Paraná, Curitiba, Brazil; ^4^Department of Information Technology, Ghent University/imec, Ghent, Belgium

**Keywords:** animal well-being, automation, digital agriculture, ethics, internet of things, sensors, smart farming, technology

## Abstract

Research and development of Precision Livestock Farming (PLF) is booming, partly due to hopes and claims regarding the benefits of PLF for animal welfare. These claims remain largely unproven, however, as only few PLF technologies focusing on animal welfare have been commercialized and adopted in practice. The prevailing enthusiasm and optimism about PLF innovations may be clouding the perception of possible threats that PLF may pose to farm animal welfare. Without claiming to be exhaustive, this paper lists 12 potential threats grouped into four categories: direct harm, indirect harm via the end-user, via changes to housing and management, and via ethical stagnation or degradation. PLF can directly harm the animals because of (1) technical failures, (2) harmful effects of exposure, adaptation or wearing of hardware components, (3) inaccurate predictions and decisions due to poor external validation, and (4) lack of uptake of the most meaningful indicators for animal welfare. PLF may create indirect effects on animal welfare if the farmer or stockperson (5) becomes under- or over-reliant on PLF technology, (6) spends less (quality) time with the animals, and (7) loses animal-oriented husbandry skills. PLF may also compromise the interests of the animals by creating transformations in animal farming so that the housing and management are (8) adapted to optimize PLF performance or (9) become more industrialized. Finally, PLF may affect the moral status of farm animals in society by leading to (10) increased speciesism, (11) further animal instrumentalization, and (12) increased animal consumption and harm. For the direct threats, possibilities for prevention and remedies are suggested. As the direction and magnitude of the more indirect threats are harder to predict or prevent, they are more difficult to address. In order to maximize the potential of PLF for improving animal welfare, the potential threats as well as the opportunities should be acknowledged, monitored and addressed.

## Introduction

The agricultural digital revolution is creating rapid changes in all types of farming. In animal farming, the term “Precision Livestock Farming” (PLF) is already well established. PLF has been defined as the management of livestock production using the principles and technologies of process engineering (1). PLF systems usually consist of sensors that may be attached to or implanted inside the animals (e.g., accelerometers, RFID rumen bolus with pH sensor) or placed in the barn (e.g., cameras, microphones, temperature loggers). Such sensors provide vast amounts of data about the animals or the environment they are living in. These data are commonly stored on a (remote) server. Artificial intelligence is increasingly used to process and analyze sensor data, for example by applying algorithms instructing a machine to take an action (e.g., modify the ventilation rate, allowing a cow to enter the milking robot) or to inform humans, usually farmers, about the condition and state of the animals and to notify them about situations that likely require a management action (e.g., artificial insemination of a cow in heat, treatment of an animal with a physical disorder).

The strengths and opportunities of PLF for improving the sustainability of animal agriculture and the animals' welfare are lauded in scholarly contributions and grant applications for research and innovative industrial developments. Claims related to the potential of PLF for improving animal welfare refer for instance to earlier and better detection of welfare issues in real time, objective and continuous monitoring of welfare indicators, automated and smart adaptations of the animals' surroundings linked to the monitoring of their condition and behavior, and the ability to focus on individual animals even in large groups (2–4). Plenty of experimental research and proofs of concept on applying PLF for improving animal welfare have been done (5, 6), but few of these systems have been commercialized and even fewer enjoy wide implementation in practice. This observation is corroborated by Abeni et al. (2019) (7), who reported that on Italian farms automated systems for monitoring locomotion problems are very rare (0.2%) compared to systems for automated estrus detection (48.6%), automated milk recording (39.4%) or even automated milking systems (3.4%). Similarly, Caja et al. (2016) (8) stated that at least 11 accelerometer-based systems for estrus detection in cows have been commercialized, compared to only two sensors for lameness detection. Consequently, scientific evidence that PLF is having a large and positive impact on the welfare of animals on commercial farms is, to the best of the authors' knowledge, still scant. In addition to the novelty of PLF systems that focus on animal welfare, there may be other reasons for the poor uptake of these commercial systems. Farmers tend to have a rather narrow definition of animal welfare which is mainly linked to the health and performance of the animals (9, 10). This may reflect limited interest in the other aspects of animal welfare that can be monitored or improved by PLF systems. Moreover, farmers generally assert that their animals' welfare status is already adequate (11, 12). Last, farmers may fear that by allowing more monitoring of animal welfare indicators they may also expose themselves and their operation to scrutiny and inspection.

Examples of early PLF systems that have been commercialized and are being increasingly adopted by the industry include automatic milking systems and pedometers for estrus detection in cows (13, 14), electronic feeding stations for sows (15), and automated weighting systems for chickens (16). Important to note is that the primary focus of these systems is not the improvement of animal welfare but rather the improvement of production efficiency and farmer quality of life. This focus alone does not suffice to ensure commercial uptake, of course. Many system requirements must be met (e.g., with regards to return on investment or user-friendliness) and the PLF system also needs to live up to expectations. Indeed, the PLF systems that are now becoming more widely adopted by famers also can provide evidence that they not only *claim* to improve production performance or farmer quality of life, but that they actually *do* so. For example, the accuracy of commercial sensors for estrus detection has been reported to be equal or superior to visual observation (17). Rotary dairies with automated technologies have been shown to have 43% higher labor efficiency and 14% higher milking efficiency (18). Automatic milking systems have been reported to reduce labor time, to increase labor flexibility, and to improve farmers' quality of life (19–24).

In contrast, the reality of whether PLF is living up to the magniloquent expectations regarding animal welfare in the livestock industry remains largely hypothetical. To date, however, claims of a beneficial effect on animal welfare in practice remain to be substantiated with hard evidence. Regardless of whether those optimistic claims will be substantiated at a future date, the aim of the present paper is simply to provide an alternative view by focusing on the potential weaknesses of PLF adoption for animal welfare, and possible threats that PLF may present. A threat concerns the possibility that something unwanted will happen, especially if a particular action is not followed. So the evidence and likelihood that these threats will become reality may differ considerably, and for some raising awareness might be the first step of a mitigation strategy. By acknowledging and addressing these potential threats, we offer counterweight to the sometimes shallow and overly optimistic beliefs regarding the beneficial effect of new technologies in order to maximize the potential of PLF to improve animal welfare under real-life, practical conditions.

Without claiming to be exhaustive, we aimed to list the potential threats of PLF for animal welfare, as well as to propose a logical categorization with the goal of facilitating their recognition and the planning of mitigation strategies. We identified 12 main threats of PLF for animal welfare, grouped into four categories: (1) direct negative effects on the animals (Threats 1–4); (2) indirect effects via the impact of PLF on the end-user (usually the farmer or caretaker; Threats 5–7); (3) a potential transformation of animal farming (Threats 8–9); and (4) a threat to the moral status of farm animals in society (Threats 10–12). Each threat is described and the likely causes and possible solutions are discussed. The threats are loosely ordered according to the expected ease or difficulty of solving them.

## Direct Negative Effects on The Animals

### Threat 1: Technical Failures Within the PLF System

PLF systems may be subject to technical failures or malfunctioning due to power cuts, software bugs, computer or other hardware breakdowns, signal transmission failures, tag losses and so on. The hardware is often vulnerable to the harsh farm environments such as high dust or ammonia concentrations and the presence of non-target animals (e.g., insects, spiders, rodents) that can damage materials or obstruct sensors. One extreme yet not unimportant threat to animal welfare is the risk that a technological malfunction could cause a barn fire. Perhaps more likely is the lack of an adequate backup plan in case of technological malfunction, especially in farms that have become highly dependent on the PLF technology for animal care and management, as for example large farms that are highly automated and under-staffed. For farmers, technology breakdown can be very frustrating and stressful, as they are often unable to solve the problem themselves and are thus dependent on external help from a specialist. The threat of technical failures has been addressed previously, as Andrade and Anneberg (2014) (25) have even identified technology breakdown as a risk factor for animal neglect in Danish pig and cattle farms.

This threat has obvious and potentially feasible solutions. First, ensure robust construction of PLF technologies before commercializing them. One could even consider a certification of PLF technologies before market introduction, as is required for technologies used for human applications such as the Electronic Health Records used for the telematic of patients (elderly, disabled, and chronically ill), smart applications for personalized healthcare monitoring, and structural health monitoring (26–28). Second, complement PLF systems with alarms that signal mal-functioning of the technology due to e.g., lack of power or internet connection. Third, ascertain that farmers have back-up plans. Farmers and caretakers should be encouraged to prepare for emergencies, e.g., by purchasing an emergency electricity generator and purchasing PLF systems only from reliable companies that offer immediate and effective 24/7 customer support.

### Threat 2: Effects of the PLF System on the Animals

The PLF system may have a direct adverse effect on the behavior, health or comfort level of the animals. Animals may find it stressful to learn to operate or adapt to PLF systems such as automatic milking or feeding systems, and if they fail to learn this swiftly they may suffer from hunger or may even be removed from the herd. Electronic feed systems may also become a competitive resource that can evoke aggression in the group, even resulting in fear and poor body condition among animals ranking low in the social hierarchy (29–31). Some technologies can also play a role in the transmission of pathogens. For example, automatic milking systems have been associated with increased transmission of pathogens resulting in higher bulk tank somatic cell counts (32). Furthermore, automated milking systems disturb the cows' natural tendency to synchronize activities and they promote zero-grazing housing systems. Sensors that are implanted inside or attached to the animals (whether using a wearable or not) may get lost, attract unwanted attention and harassment from pen-mates, or directly cause discomfort and lesions. For example, a wearable for attaching an ultra-wideband tag for tracking the locations of chickens needed to be redesigned due to behavioral changes caused by the wearable in the short term (33) and injuries in the longer term (34). Although a new design solved these problems, a novel problem emerged over time: the wearable and tag casing had attracted significant colonization with red mites. The chickens then required frequent treatment with mite deterrents (Michael Plante-Ajah 2021 personal communication). Obviously, ear tags can also cause damage to the animals' ears (35).

Little is known about the welfare effects of repeated or long-term exposure to PLF hardware such as noise, radiation or stray voltage. It is not surprising that such studies are rarely conducted prior to market introduction (and afterwards) as they tend to be expensive: they often require longitudinal monitoring of subtle signs from an adequate number of animals and herds. The distress to the animals may not be obvious to humans due to differences in sensory capacities. Further, such studies may yield unfavorable outcomes that would delay or stop the commercialization pathway. A recent study by Van Shaik et al. (36) highlights the need for such studies, however. In the Netherlands, multiple instances of unexplained adverse grouping behavior of dairy herds were reported in which the animals avoided a part of the barn. This resulted in increased standing and reduced lying, drinking and feeding time, which in turn increased the risk of lameness. A matched case-control study revealed several risk factors, many of which were characteristic of high-tech farms: the use of automatic milking systems, investigation of stray voltage with application of mitigation measures, and recent date of construction.

Solutions to these types of threats include (1) the use of a minimal number of multi-purpose sensors, (2) prioritizing sensors and PLF systems that are minimally invasive or uncomfortable, (3) wearer-driven designs with minimal size and weight, noise-production and radiations, and (4) adequate longitudinal testing of the consequences of the PLF system on the behavior and welfare of the animals under a variety of commercially relevant conditions.

### Threat 3: Poor External Validation of the PLF System

The algorithms of the PLF system may perform poorly under real-life conditions, possibly resulting in unreliable alerts, with both false positives and false negatives, or in inaccurate monitoring. When management actions and decisions are being driven by false or incomplete information, animal discomfort or harm can result. As a consequence, the caretaker may experience frustration, which can indirectly impact the human-animal relation and hence the performance and welfare of the animals (37, 38).

The possible causes of low accuracy of the algorithms are multiple and diverse. Algorithms are typically developed by making use of training data. If the training data are poor, the algorithms are also likely to perform poorly. This is referred to as “garbage in, garbage out”. For example, an algorithm will perform poorly if it is developed on the basis of “gold” standards that are flawed (e.g., training data has poor validity or reliability and therefore includes animals whose behavior or condition have been labeled incorrectly). Over-fitting is a well-known problem in artificial intelligence applications where the model has a high accuracy with the training data (high internal validity), but performs poorly in a new situation (poor external validity). When the training dataset is different and less variable than the conditions where the system will be used, the external validation of the system is clearly inadequate. A well-known example of “over-fitting” is a student assignment to create an AI algorithm that could differentiate pictures of a wolf from pictures of a husky. The resulting algorithm showed high accuracy with the training data, but appearances were deceiving: the system had learned to categorize pictures with snow as a wolf and pictures without snow as a husky because nearly all pictures of a wolf happened to be taken in the snow, while the husky pictures rarely had snow in the background (39). This example illustrates how AI systems don't actually understand the task at hand: they are black box systems that give no information about the characteristics used for performing the task. An additional complicating factor is the different types of variance within animal production systems. For example, several commercial PLF technologies have been developed for dairy cattle welfare assessment, but the majority of them have only been validated on adult cows, not on calves and heifers (40).

PLF systems are commonly claimed to be objective, in contrast with human observers performing assessments or giving scores which may be subject to conscious or unconscious rater biases. If human biases are present in the training data, however, AI may reproduce and reinforce these biases while giving them the appearance of objectivity. A famous example of such an “algorithmic bias” is Amazon's AI recruitment tool that matched job applicants with vacancies. The AI training was based on submitted résumés, which applicants had and had not been hired, and which were judged to be performing well in their job. Upon closer examination, these training data appeared to show a bias against women. Most résumés came from men and male candidates were favored over women. This human gender-bias was faithfully reproduced in the AI recruitment tool (which was later scrapped (39)).

The problem of poor validation can be addressed by (1) setting quality guidelines for proper validation and performance testing of PLF systems; (2) training and testing PLF systems in the full range of conditions under which they may be used prior to market introduction or at least transparently reporting the conditions under which it has and has not been validated; and (3) regulations or contracts that hold the manufacturer liable in case the end-user suffers damage or losses due to a poor performance of the PLF system due to inadequate validation. These recommendations conflict with the economic pressure on technology providers to quickly introduce technological innovations at a competitive price. In practice, only 5% of the human wearables appear to have been formally scientifically validated (41). External validations trials are available for only 14% of the retailed PLF systems for dairy cow welfare assessment (40), and only 23% of publications related to PLF in pigs were properly validated (42).

### Threat 4: Focus on the Measurable Rather Than Meaningful

What can be measured by PLF systems is not necessarily what is most meaningful to measure for animal welfare. The available PLF measures may not be the most important, valid, sensitive or complementary set of indicators for assessing animal welfare or for identifying which animals require attention by the farmer. Although the booming PLF research and development is often focused more on improving animal welfare than on increasing production (6, 40, 43–51) not many innovations proceed from the research and development phase to becoming commercialized, let alone being adopted by animal farmers. On the contrary, the PLF-systems that are being brought to market and adopted by farmers are those that focus on production efficiency and farmer quality of life.

Current animal welfare assessment strategies commonly rely on short farm visits during which health issues and resource-based indicators are scored, while behavioral observations tend to be limited to short time windows. Although PLF has great potential to complement and improve such protocols, the proportion of PLF systems focused on animal welfare that proceed from the research and development phase to being taken up by the livestock sector at present is very limited (43) as well as biased. Even current farm animal welfare assessments suffer from a one-sided focus on physical or behavioral problems, rather than on positive indicators or measures of the animals' affective state. The use of PLF may amplify this bias. The animal welfare measures that researchers and developers consider to be relevant may not correspond with what manufacturers think could be profitably marketed nor what end-users perceive as good value for money and would thus actually purchase. Even if the willingness of famers to purchase PLF systems that focus on animal welfare would increase, it is likely that these would tend to be measures that are relatively easy to monitor, measures that document the most common problems and how they are usually expressed, and those that the farmer considers to be relevant for animal welfare (provided they are neither intrinsic to the production system nor very difficult to solve). Unusual animal welfare problems or problems that are expressed in an uncommon way, due to specific environmental conditions or individual idiosyncrasies, may thus go unnoticed as the PLF systems will not recognize these as problematic. Farmer beliefs play an important role in the adoption of technology (52). This explains for instance the higher adoption potential of technologies that focus on animal welfare problems as compared to technologies that focus on positive welfare measures (53).

Algorithms may also be trained to consider the average state as good and may thus signal all deviations from the average as potentially alarming—even when the average state may not be optimal for the welfare of the animals. As mentioned above, farmers already tend to have a rather narrow, health- and production-centered view of animal welfare (10). Commercialization of farmer-pleasing PLF systems may exacerbate this shift of focus from measures motivated by a need for assessing animal affective state and emotions to the more measurable and quantifiable indicators of physical health and selected behavioral expressions. Such a shift in how animal welfare is measured and thus defined may have far-reaching and perhaps even counterproductive effects on the welfare of farm animals. Indeed such a health-based definition of animal welfare favors more restrictive (intensive) housing systems that highly rely on human inputs, control and oversight to safeguard the physical integrity of the animals rather than the less restrictive but more natural (extensive) housing systems that offer the animals more psychological opportunities and self-control (54).

Ways to address this threat are (1) to critically evaluate the nature and comprehensiveness of the animal welfare data that a PLF system is gathering and processing; (2) to conduct sensitivity analyses, and if needed, use a disclaimer for animal welfare aspects that cannot be documented by the available data or instigate efforts to address such gaps (which likely will concern indicators of the animals' affective state); (3) to refrain from claiming that PLF can assess overall animal welfare or identify all animals that suffer welfare problems; and (4) to complement PLF data with manual animal welfare assessments carried out by an experienced human. To address the threat that out-of-the-ordinary (expressions of) welfare problems could go unnoticed by the PLF systems, multiple generic or so-called “iceberg” indicators of animal welfare (55, 56) could be included. Additional ways to detect anomalies could be to assess individuals that deviate from the herd-average as well as to assess changes within individual animals over time.

## Indirect Effects VIA the Impact of PLF On the End-Users

### Threat 5: Over- or Under-Reliance on PLF

PLF may also indirectly pose a threat to the welfare of farm animals by affecting the people that interact with the animals: the farmer or the caretaker. Animal welfare may suffer if the end-user shows either blind trust (over-reliance) or not enough trust (under-reliance) in the PLF system. End-users rarely understand how the PLF system works and what its limitations are, while the PLF engineer developed it from a primarily technical perspective that often neglects externalities. This mismatch may impede the end-user from using the machine as intended. The few studies in which this threat has been investigated suggest low farmer confidence in PLF-systems. For example, Hogeveen et al. (57) reported that only 21% of the disease alerts (given when tags indicated a more than 30% reduction in activity, eating or lying time) successfully activated dairy farmers to visually check the cow. They found that farmers were more likely to perceive an alert as true and to follow it up with a visual check of the animal if the number of alerts per day was manageable (<20 alerts/d), for cows in the transition period (known to be a high risk period), and when alerts arrived during the work-week instead of on the weekend. Information overload and a perception of poor accuracy or minimal usefulness of the PLF alerts seemed to reduce the likelihood that farmers would act upon the alerts. Over-confidence on the PLF system may also occur, although little evidence for this exists. One possible example, although not related to animal welfare assessment, is a report where 15% of the participating dairy farmers relied exclusively on estrus detection sensors when deciding when to inseminate (58).

Neither a lack of trust nor a blind trust in the PLF system is desirable. A lack of trust will likely result in frustration among the end-users, who in this case experience little added value from the system they are presumed to be using and may also have invested in. This in turn may negatively affect the human-animal relation and implies that the potential added value of the PLF system is not being utilized. In the case of blind trust, the end-users may no longer be vigilant for signs of animal welfare issues and may even feel less responsibility for the welfare of the animals. The threat is that false positive alerts will wrongly be acted upon, whereas false negatives will remain unnoticed.

This threat can be addressed by (1) ensuring the PLF system performs accurately under commercial conditions, provides information that is useful to the end-user, and is user-friendly; and (2) by training and supporting end-users to make correct use of the system.

### Threat 6: End-User Spends Less (Quality) Time With Animals

This threat concerns the amount of time farmers spend with their animals and the nature and quality of the human-animal interactions. Currently adopted PLF systems often focus on reducing labor. When PLF systems successfully reduce the farmer's workload, the effect on the animals depends on how the additional free time is used. In case of strong economic pressure, that free time may be used primarily to reduce overall labor costs through contracting fewer working hours or to expand the farming activities (e.g., by increasing the herd size without increasing the number of laborers). In theory, extra free time could be used by the farmer for spending more time with his animals, for observing, checking or interacting with them, and for providing more care to those who need it. In practice, though, it is more likely that the unrelenting quest to reduce production costs and to remain economically competitive will require an increase in herd size without increasing the number of workers—either to repay the investment in the PLF or for another economic activity. In such a case, the PLF system will result in less time spent between humans and individual animals on the farm, resulting in animals that may become less habituated to people and decreased human attention to individual animals. Concerns have already been raised regarding the extent to which PLF could redefine the farmers' attitude and notion of care toward animals (51, 59).

Besides negatively affecting contact time spent per animal, the nature of the human-animal interactions may also be affected (51). The type of interactions that can be taken over by PLF systems are often neutral or positive, such as feeding, milking or observing the animals. The type of interactions that are harder to automate and are thus carried out by the farmer tend to be the more negative interactions such as mutilations, vaccinations or moving the animals. This may negatively impact the human-animal relation, leading to an increase in the amount of fear and stress experienced by the animals when handling is performed. This may turn into a negative spiral in which farmer's job satisfaction is reduced and could in turn further worsen how the farmer interacts with the animals. Less time spent per animal may also reduce the farmer's knowledge of the individuals in the herd, their personalities and peculiarities, and therefore their ability to detect problems and anomalies. The PLF system will mainly alert the farmer to (problem) animals that require more immediate attention. The other animals may remain rather invisible, with unrecognized identities and personalities, possibly resulting unintendedly in them being seen more as an “outgroup” and thus less worthy of concern, dignity and respectful treatment (60).

This notion relates to the long-known contact hypothesis, which states that the amount of interaction and care for living beings positively affects our concern for them and our attitude toward them (61). Various studies support this hypothesis. Weatherill (62) and Ascione (63) documented that personal interactions with animals provide the best opportunity for bonding and empathic response. Morris et al. (64) showed that people who have never kept any animals recognize less capacity of animals to experience emotions. TomaŽič (65) concluded that children who self-report direct experience with amphibians report less fear and disgust toward them. Randler et al. (66) concluded that (respectful) physical contact during school practicals reduces disgust and fear of wood louse, snail and mouse in children. Strong evidence for the big impact of direct experience and interaction with individual animals was also provided by an experiment by (67). They showed that a 2-h clicker-training practical involving many personal interactions and contact with individual chickens had a profound impact on animal and veterinary science students' opinion concerning chicken intelligence, personality and ability to learn and to feel emotions. Similarly, the level of attachment or detachment of farmers toward their animals seems to be affected by the frequency, intensity and intimacy of their interactions with animals (68). Of course, the significance of this threat differs between farm animal species and the amount of contact amongst caretakers and individual animals, which is typically much less for e.g., broiler chickens than for dairy cattle.

Thus, if PLF systems result in more free time it is advisable that the farmer spends some of that time with individual animals and preferably in a positive manner. Perhaps PLF, by decreasing time consumption in an array of activities, can potentiate the amount and the quality of farmer attention to animals, creating possibilities for closer emotional contact between caretakers and at least a few animals, e.g., those that are in greatest need of care, and such contact may reduce objectification of the other animals under his care as well. The influence of PLF, the intensification of animal production and the amount and type of labor on farmers' attitude and behavior toward animals deserves more investigation. Creating an environment of care, concern and respect for animals may be more important than commonly acknowledged in animal farming. Finally, there is a need to develop and apply PLF that focus on assessing the human-animal relationship.

### Threat 7: End-User Profile and Skills

This threat concerns a longer-term change in the animal caretakers' profile and skills. Reliance on PLF for detecting animal welfare issues or animals in need of attention may reduce the caretakers' own skills and efforts to detect such issues. This may be exacerbated by a shift toward a more technology-centered farm staff profile. Whether such a shift will be accompanied by diminished attention for the animals remains to be seen, as animal orientation seems to be a rather stable personality trait that cannot be readily modified (69, 70). The threat is that if caretakers become less skilled in animal care activities and less animal-oriented, the risk of inappropriate attitudes and behaviors toward animals may increase. Such traits would represent an increased risk of not noticing animal welfare problems when PLF systems fail. There are no easy solutions to prevent this from happening. The main recommendation is to ensure that both technology skills and animal orientation are addressed in stockpersons' training and included as recruitment criteria. Overly stringent selection criteria, to demand both technology skills and animal orientation will further narrow the already limited supply of skilled farm laborers (71–73). On the other hand, a reduction of repetitive and physically-intense activities which may be performed by automated systems coupled to opportunities for positive interactions with animals may revive the interest for rural work.

## Indirect Effects VIA Potential Transformation of Animal Farming

### Threat 8: Housing and Management Adapted to PLF Instead of Animals

Animal welfare threats that relate to the impact of PLF on either animal farming in general or the moral status of farm animals in society are particularly difficult to prevent or address. No one can predict how exactly PLF will affect animal production practices or the status that society will give to farm animals. Nevertheless, some trends appear to be self-evident and are likely to be nearly impossible to counter or prevent. For example, as PLF becomes more popular and a standard part of modern farming, it seems inevitable that the housing and management of animals will become increasingly adapted to the PLF systems, possibly at the expense of the animals' welfare. First, in production systems that are commonly associated with better animal welfare, such as small-scale, extensive or outdoor systems, certain PLF technologies are already known to be less profitable, more difficult or even impossible due to the lack of (wireless) internet connection, lack of a power supply, the fact that the area to cover is too large, or that the costs per animal are too high. Moreover, an optimal environment for PLF performance (e.g., the best light characteristics for the accuracy of camera-based PLF) may not be the optimal environment for the animals. Many PLF systems require animals to be identified and filtered out from the background environment in which they live. This tends to be easier and more accurate if the background environment, considered “noise”, that needs to be filtered out is homogeneous, besides being clearly distinct from the animals. This need will likely favor barren, sterile housing systems in which the welfare of the animals is compromised. Such environments limit the animals' choice, agency, control, opportunities for distraction and stimuli to perform species-specific behaviors. This threat can be mitigated by setting animal-centered housing requirements that accommodate species-specific behavioral needs and allow animal agency. The economics of scale may also dictate that certain PLF systems, such as those based on camera or acoustic surveillance, are more cost-efficient if the animals are housed in one large group instead of many different groups. This may be mitigated by encouraging the development and uptake of PLF systems that are specifically designed to facilitate animal management in small-scale or extensive systems (74).

### Threat 9: Facilitation of Intensive Systems

Similarly, PLF may facilitate further intensification because the current focus of PLF is often on production efficiency, farm profitability and labor savings. That focus is reinforced by the economics of scale which dictate that the per capita cost of PLF is lower on large farms. This increases the likelihood of adopting PLF in large-scale intensive systems. This relation has been corroborated by Cargiui et al. (75) who found that herd size is a leading determinant of adoption of PLF. Similarly, Abeni et al. (76) found that the use of automated technology on dairy farms was associated with large herd size, high milk yield and high cow-to-worker ratio. Steenveld and Hogeveen (76) also reported that the number of labor hours per cow per week was lower in dairy farms with conventional milking systems with sensors than farms without sensors.

Although the link between intensification or herd size and animal welfare is complex and affected by many factors (e.g., managerial skills, rate of herd expansion, ratio of caretakers to animals), when a relationship is found with animal welfare aspects, it is often a negative one. It has been reported, for example, that mortality (77) and prevalence of infectious diseases (78–80) generally increase with herd size. Herd expansion has been associated as well with increased mortality and reduced animal welfare (81). We need to be on our guard that PLF does not become yet another advancement to further production efficiency in which animals pay the price. As recognized for many years, there is a cost to be paid for a blinkered drive toward ever-cheaper animal products, and that cost is paid by the animals (82). In order to mitigate this threat the uptake of PLF systems that counteract the animal welfare risks associated with large-scale, intensive production (e.g., difficulty of keeping track on animal welfare problems of all individuals) could be promoted.

Further intensification may also amplify the disconnection and alienation between conventional livestock production and the public's calls for more “natural” production methods. This may thus endanger the social license to produce. A more indirect and subtle threat is that industrialization typically requires large infusions of capital that only integrators or retail corporations can afford. As a consequence, farms are increasingly owned by integrators and retail firms, who are empowered to dictate prices and production practices. When the farmer is no longer in charge of the farm, an “erosion of the ethical attitudes and behavior of farmers” could result (83). Changes in industrial agriculture and constrained choice and agency increase the likelihood that farmers will consider unethical behavior.

## Indirect Effects by Affecting the Moral Status of Animals in Society

### Threat 10: Increased Speciesism

PLF may not only affect the attitude and behavior toward animals of the people directly involved in the animal industry, but also of society at large. Currently, one can only speculate about the precise nature of these influences because PLF is only now breaking through, and to the best of the authors' knowledge, studies that have investigated such societal effects are still lacking. One concern is that unequal adoption of animal welfare PLF among the different types of farm animals may lead to, or further exacerbate, differences in the moral status allocated to them by the animal industry and by society. Indeed, the (speed of) uptake, the opportunities and the type of PLF systems for the various farm animal species are likely to be determined by factors such as economics, animal size and body conformation, or housing system, rather than the priorities with regard to animal welfare (number and severity of animal welfare issues). For example, animal welfare PLF seems more readily brought into market and adopted by dairy cattle farmers than by producers of chickens, fish or rabbits: these smaller individuals represent a smaller monetary value per animal (51). The type of PLF system per species also differs strongly: adoption of sensors that are attached to individual animals seems much more feasible for dairy cattle as compared to chickens or even pigs (51). Differences between species in care and moral considerations for individual animals may thus be reinforced by greater use of PLF systems that provide data about the individual rather than the entire group (e.g., dairy cattle as compared to poultry). Interpreting group level data and taking appropriate actions in response to that data can indeed be more challenging than for individual-level PLF data that give information about the specific condition and needs of individual animals. Certain management decisions made at group level may also be detrimental to the welfare of individuals whose needs differ from others (51). Such differences among animal species in the degree and type of PLF used for monitoring or improving their welfare can be expected to lead to unjust differences in moral status, concern and care amongst animals from different species, breeds, lineages or even life stages, and hence to speciesism.

### Threat 11: Increased Animal Instrumentalization

**Figure 1:**
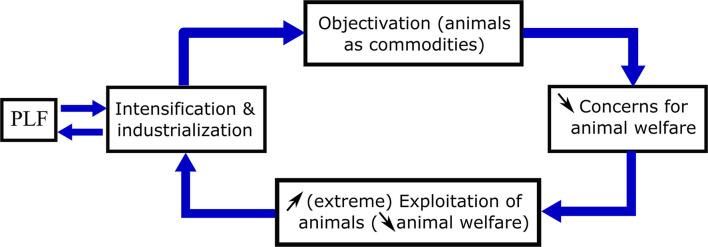
Reciprocal strengthening links between two closed loops: the mutually reinforcing link between PLF and the intensification of animal farming (loop 1) and between increasing intensification of animal farming and reduced concern for animal welfare (via objectification of animals; loop 2).

The reciprocal strengthening effects between PLF and the intensification of animal production (threat 9), may have broader animal welfare consequences because of their impact on the moral status of farm animals in society. Further intensification and industrialization of animal farming, where vast numbers of farm animals are hidden away indoors in large farms not visible to the public, makes it even more difficult for the citizen to view these animals as individuals with different personalities and characteristics. This facilitates the objectification of farm animals, which are seen and treated as commodities, not only by farmers but also by citizens (59, 84). Instrumentalization of animals decreases the concern for their welfare, providing the social license to exploit even more animals in a more extreme way for human interests. This in turn will facilitate further intensification of production systems in the livestock industry, closing the loop of positively reinforcing effects (Figure 1). If this closed loop of self-strengthening effects is not restrained or halted, the consequences for farm animals could be severe.

### Threat 12: Promotion of Animal Consumption and Harm

**Figure 2:**
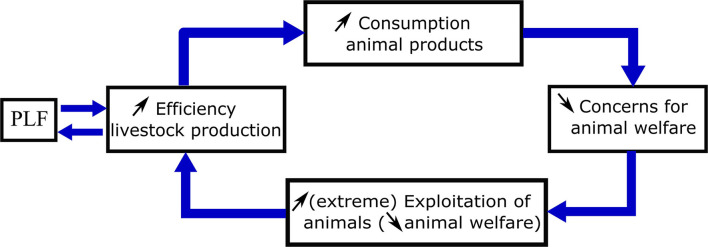
Reciprocal strengthening links between two closed loops: the mutually reinforcing link between PLF and the efficiency of livestock production (loop 1) and between increasing the efficiency of livestock production and reduced concern for animal welfare (*via* the increased consumption of animal products; loop 2).

PLF may facilitate an additional variant of a closed loop of positively reinforcing effects that could be detrimental for the welfare of farm animals (Figure 2). As argued above, PLF systems currently adopted in the animal industry are often focused on increasing production efficiency, and PLF systems are more likely to be adopted by those for which production efficiency is of crucial importance for their (international) competitiveness. These mutually reinforcing influences, leading to a continuous quest to increase the efficiency of animal production, implies that animal products will become more affordable to an ever-increasing number of people worldwide. There are indications that the consumption of animal products in itself diminishes the concern for animal welfare and thus may reduce the moral status of animals in society. Indirect evidence for this link is provided by reports that meat consumption is linked with less positive attitudes toward animals (85–87). More direct evidence of a link between meat consumption and a diminished moral concern for animals is provided by the experiment by (88). They surveyed people who were blinded to the fact that they had been subjected to two experimental treatments: shortly before filling out the survey half the participants had been given a meat snack whereas the other half of the participants had been given a plant-based snack. Those who ate the meat snack gave a significantly lower score to the extent a cow deserves their moral concern and indicated significantly fewer animal species, from a list of 27 species, for which they feel moral concern. The authors interpreted these results as evidence of cognitive dissonance. Eating animal products while realizing these products are derived from sentient beings that are exploited by the livestock industry against the interests of the animals themselves is thought to potentially cause mental friction and stress. One common way by which people relieve this friction is to downplay the ability of non-human animals to experience emotions compared to humans, and to inflate the difference in moral status between humans and other animals, so-called “infra-humanization” of animals (60).

## Conclusion

The digital revolution will continue to have a far-reaching impact on animal farming. It is not yet clear whether the animal will be the victim or the beneficiary of this revolution (89). Many PLF technologies that focus on monitoring or improving animal welfare are being developed and researched. The potential benefits and opportunities of these technologies are being praised, with a general tenor of euphoric optimism about their future effects on animal welfare. To date, however, only a small proportion of these technologies have been brought to market, and an even smaller proportion is being adopted by farmers. The true PLF breakthrough is not happening in regard to animal welfare, but rather in the areas of production efficiency and laborer quality of life. Some studies have been conducted that show that on these fronts, some of these technologies do live up to expectations. For PLF technologies that focus on animal welfare, however, it remains unclear whether they will ever be widely adopted commercially and whether they will have the hoped-for beneficial effect for the animals.

The introduction of PLF represents many exciting opportunities while it may also pose considerable risks and threats for the animals. Without claiming to be exhaustive, this paper indicates 12 ways in which PLF technologies may harm the animals directly or indirectly: they may affect the animals, the farmers, animal farming in general, and even society as a whole. The more direct the threat to animal or farmer, the easier and more feasible the suggestions about how to prevent and resolve these threats. The current market form—an open international market that prioritizes economic interests even at the expense of the interests of the animals concerned—often means that less animal-friendly production methods achieve a competitive advantage. In practice, the present recommendations to address the direct threats (i.e., that PLF technologies should be thoroughly externally validated, longitudinally tested for adverse effects on the animals and for robust functioning in a wide variety of commercial conditions before they are brought to market, and the provision of adequate customer support, advice and training) may inflate the costs so much that the technology becomes too expensive for commercial adoption. Alternatively, manufacturers that introduce seemingly similar technology sooner and without such thorough testing may gain a competitive edge.

The indirect potential threats are even harder to avoid or mitigate. Even if PLF with a focus on animal welfare were to be widely adopted, farmers would also need to prioritize animal welfare by considering species-specific behaviors and individual emotional states in addition to the more commonly-valued indicators of physical health and performance. It is also up to the farmers to have an appropriate level of trust in the data generated by the PLF and to show willingness, provided they are empowered, to take corrective action. It remains to be seen how PLF will contribute to the changes occurring in animal farming in general, and how this can influence farmer, consumer and citizen attitudes and moral concern for animals.

Rather than assuming that PLF will, by itself, benefit animal welfare in accordance with the often honorable intentions of the PLF researchers and developers, scientists should continue to assess the effects of PLF on animal welfare via independent multi-disciplinary scientific monitoring on all levels: individual animals, farms and society. The agricultural and economic reality may pull in another direction than the original intentions.

## Author Contributions

FT, CM, and SB contributed to conception of the review. FT wrote the first draft of the manuscript. CM wrote sections of the manuscript. SB drew the figures and inserted the reference list. All authors contributed to manuscript revision, read, and approved the submitted version.

## Funding

ILVO pays for open access publication fees.

## Conflict of Interest

The authors declare that the research was conducted in the absence of any commercial or financial relationships that could be construed as a potential conflict of interest.

## Publisher's Note

All claims expressed in this article are solely those of the authors and do not necessarily represent those of their affiliated organizations, or those of the publisher, the editors and the reviewers. Any product that may be evaluated in this article, or claim that may be made by its manufacturer, is not guaranteed or endorsed by the publisher.
